# Current evidence for the hepatoprotective activities of the medicinal mushroom *Antrodia cinnamomea*

**DOI:** 10.1186/1749-8546-8-21

**Published:** 2013-11-01

**Authors:** Patrick Ying-Kit Yue, Yi-Yi Wong, Kay Yuen-Ki Wong, Yeuk-Ki Tsoi, Kelvin Sze-Yin Leung

**Affiliations:** 1 Department of Biology, Faculty of Science, Hong Kong Baptist University, Hong Kong, SAR, China; 2 Department of Chemistry, Faculty of Science, Hong Kong Baptist University, Kowloon, Hong Kong SAR, China

## Abstract

*Antrodia cinnamomea* (AC) is an endemic mushroom species of Taiwan, and has been demonstrated to possess diverse biological and pharmacological activities, such as anti-hypertension, anti-hyperlipidemia, anti-inflammation, anti-oxidation, anti-tumor, and immunomodulation. This review focuses on the inhibitory effects of AC on hepatitis, hepatocarcinoma, and alcohol-induced liver diseases (*e.g.*, fatty liver, fibrosis). The relevant biochemical and molecular mechanisms are addressed. Overall, this review summarizes the hepatoprotective activities *in vitro* and *in vivo*. However, there is no doubt that human and clinical trials are still limited, and further studies are required for the development of AC-related products.

## Introduction

**Figure 1:**
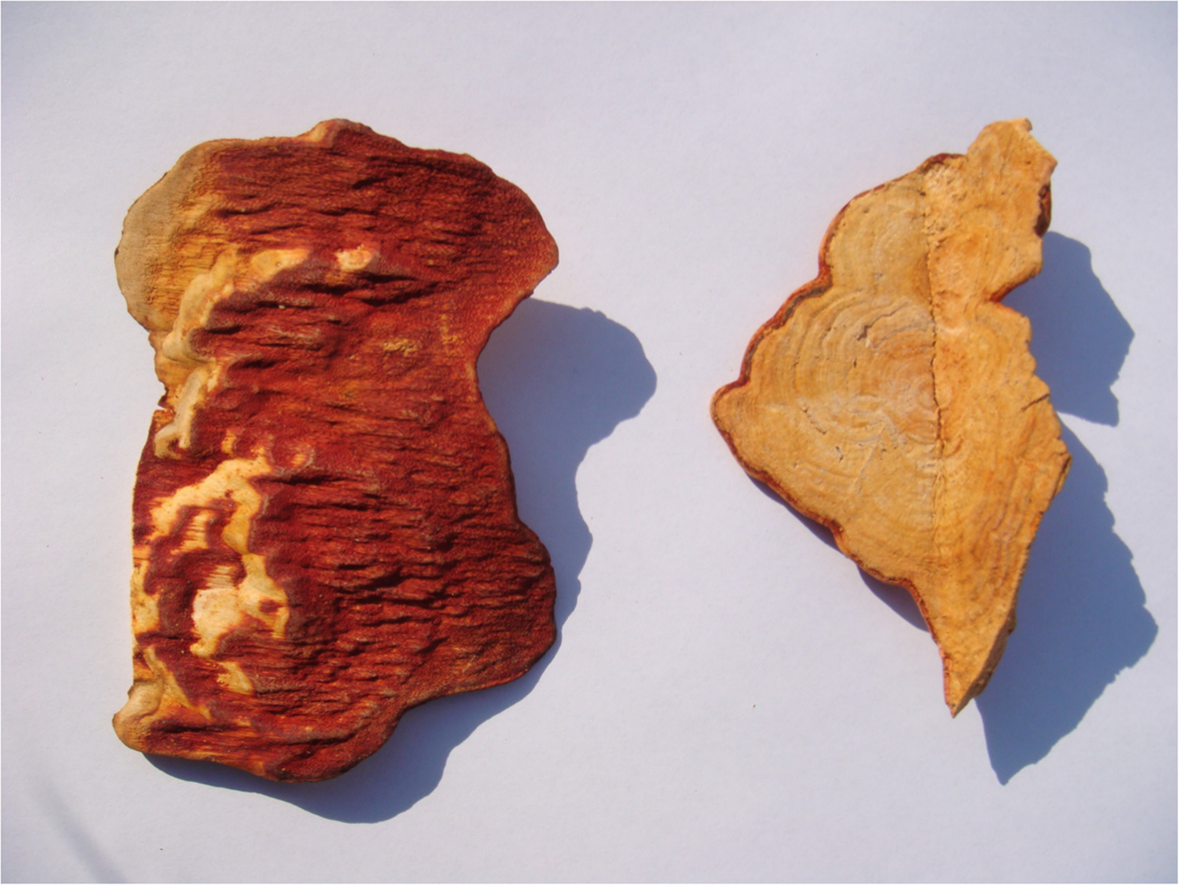
**Morphological appearance of *Antrodia cinnamomea*.**

*Antrodia cinnamomea* (AC) (*niu-chang-chih* in Chinese) is a medicinal mushroom (Figure 1) endemic to Taiwan. It has been well-known for its medicinal properties, particularly with regard to liver complaints [1], since its first identification as a new species by Zang and Su in 1990 [2]. Among its diverse pharmacological activities, the evidence for hepatic protection is the most recognized and the strongest. Studies have shown that AC can inhibit hepatic tumor growth and retard the progression of hepatitis [3,4]. Although AC is currently used as a food supplement, the Food and Drug Administration (FDA) has not approved any AC extracts or purified compounds for clinical applications. Some AC products are claimed to protect the liver against food and drug intoxication, especially alcohol-induced liver damage, maintain hepatic homeostasis, or both. This article reviews the current evidence for the hepatoprotective properties (such as effects on hepatitis, cirrhosis, liver cancers, and alcoholic damage) of AC extracts and active compounds.

## Effects on hepatitis

### General background of hepatitis

Hepatitis is one of the top ten causes of death worldwide (more than one million deaths per year), and is mainly classified into hepatitis A, B, and C, involving different types of viruses, epigenetic and genetic alterations, pathological progressions, and clinical treatments [5]. Hepatitis A is an acute infectious liver disease caused by hepatitis A virus (HAV). It is transmitted by a fecal-to-oral route, and mainly occurs as outbreaks from fecal contamination of food or water sources or direct contact with carriers. It has no clinical signs or symptoms in most infected people, and most cases recover within 1–2 months [6]. Hepatitis A causes the least problems among the various types of hepatitis, and its incidence rate has been diminishing in recent years, probably through improved hygiene [6]. Hepatitis B and C are acute and chronic infections caused by hepatitis B virus (HBV) and hepatitis C virus (HCV), respectively, and are more serious. HBV and HCV belong to the Hepadnaviridae and Flaviviridae, respectively. HBV is a DNA virus that can integrate into the host genome after infection, while HCV is an RNA virus that has no such ability [6]. According to a worldwide epidemiological evaluation, at least 400 and 170 million people are chronically infected with HBV and HCV, respectively [7,8]. In fact, these viruses persist in the liver in about 80% of infected people [9], and such chronic viral infections may be associated with chronic inflammation that leads to scarring of the liver tissues and cirrhosis [10]. Upon infection, HBV and HCV interact with host hepatocytes, impair cell metabolism (*e.g.*, induce cell death) [11], modulate signaling pathways of protein synthesis (*e.g.*, produce viral proteins and enzymes) [12,13] and immunity (*e.g.*, synthesize and place viral antigens on host cell surfaces) [14], and develop immortalization (*e.g.*, modulate surface markers). Ultimately, 1–5% of cirrhotic livers develop hepatocellular carcinoma (HCC) [15].

### Anti-hepatitis activity

Inhibition of viral proteases, which are crucial for viral replication, can interrupt viral protein maturation. Antrodins (A to E) and their metabolic analogues selectively inhibited HCV NS3/4A protease activity [3]. Among these, antrodin A (the major *in vivo* metabolite of antrodin C) showed the highest potency with an IC_50_ of 0.9 μg/mL, while antrodin B showed lower potency with an IC_50_ of > 100 μg/mL. Moreover, polysaccharides isolated from fruiting bodies and cultured mycelia of AC inhibited HBV replication activity [16]. After an HBV-producing cell line (MS-G2) was treated with the AC-extracted polysaccharides, anti-hepatitis B surface antigen (HBsAg) and anti-hepatitis B e antigen (HBeAg) were reduced [16]. Moreover, the AC-extracted polysaccharides (50 μg/mL) were found to be more effective than interferon-α (IFN-α) (1000 U/mL) for HBsAg and HBeAg inhibition [16]. Huang et al. [17] showed that 2,2′,5,5′-tetramethoxy-3,4,3′,4′-bis(methlylene-dioxy)-6,6′-dimethylbiphenyl (50 μM) could suppress the HBsAg and HBeAg levels in a wild-type HBV-producing cell line (ES2) within a non-toxic range and in a dose-dependent manner. These findings indicate that AC can effectively attenuate hepatitis virus-induced damage by inhibiting essential viral enzyme activities and antigen production.

## Effects on HCC

### Pathogenesis of HCC

Liver cancer comprises diverse hepatic neoplasms, including HCC, cholangiocarcinoma, hepatoblastoma, and hemangiosarcoma [18,19]. Among these, HCC is the primary malignant hepatic cancer worldwide, the fifth most common cancer, and the third leading cause of cancer death around the world [20-22]. The incidence and mortality of HCC are both increasing [23-26]. HCC arises in the context of chronic viral hepatitis (*e.g.*, HBV, HCV, and HDV infection), cirrhosis, alcoholism, toxin or drug poisoning (*e.g.*, aflatoxins), and metabolic disorders (*e.g.*, diabetes, non-alcoholic fatty liver disease, and hereditary diseases) [27]. Recently, geographical differences in HCC incidence have been related to the changing distribution and natural history of HBV and HCV infections [28]. The World Health Organization (WHO) reported that HBV is a human carcinogen, and that an estimated 300–400 million individuals are chronically infected with HBV worldwide [29]. Moreover, a large number of carriers fail to eliminate the virus, and progress to chronic viral infection. Continuous hepatic inflammation can lead to chronic hepatitis and cirrhosis. The incidence of HCC in chronically HBV-infected or cirrhotic individuals is 100- and 1000-fold higher, respectively, than in uninfected individuals [27,30]. For toxin-induced HCC, ingestion of aflatoxin-B1 (a fungal toxin classified as a type I carcinogen by the International Agency for Research on Cancer (IARC) and produced by the molds *Aspergillus flavus* and *Aspergillus parasiticus* found in contaminated food) increased the risk of HCC development [31,32]. Aflatoxin-B1 also induced mutations in codon 249 of the *p53* tumor suppressor gene and the *RAS* oncogene [33-36]. Alcohol, which is also classified as a type I carcinogen, is another critical HCC risk factor [31,32]. Alcohol activates monocytes and induces pro-inflammatory cytokine production, and the subsequent increase in endotoxins activates Kupffer cells to release chemokines and cytokines (*e.g.*, tumor necrosis factor (TNF-α), interleukin-1β (IL-1β), and interleukin-6 (IL-6)). The resulting oxidative stress activates stellate cells to synthesize large amounts of collagen, leading to fibrosis, cirrhosis, and ultimately HCC [37-39]. Apart from these etiological agents, inflammation, oxidative stress, genetic alternations, activation of oncogenes, inactivation of tumor suppressor genes, genomic instability, and overexpression of growth and angiogenic factors are related to the pathogenesis of HCC [31,40].

Angiogenesis plays an important role in the pathogenesis (*i.e.*, tumor growth, invasion, and metastasis) of all types of solid tumors, including HCC [41]. As a hypervascular tumor, HCC develops and progresses from a small, avascular, and well-differentiated HCC to a large, highly vascular, and poorly differentiated HCC within a short period [42]. The expanded endothelium provides a channel for HCC cells to enter the blood circulation system and metastasize to different tissues. During tumor angiogenesis, angiogenic factors (*e.g.*, vascular endothelial cell growth factor (VEGF), fibroblast growth factor-2 (FGF-2), angiogenin, or angiopoietins) and their receptors are produced by tumor cells and endothelial cells (ECs) [43,44]. Through VEGF signaling, the most common and potent angiogenic signaling pathway, ECs express VEGF receptors (VEGFR-1 and -2), receive signals from tumor-generated VEGF, proliferate, migrate toward tumor tissues, and form functional neovessels [43,44]. Moreover, these angiogenic factors induce gene transcriptional, biochemical, and enzymatic activities, such as extracellular digestion of extracellular matrix by matrix metalloproteinases (MMPs), cell cycle progression, and production of bioactive molecules (*e.g.*, angiogenic factors, cytokines, and chemokines) [43,44]. As a result, both tumor cells and ECs are activated in a feedback mechanism, thereby facilitating tumorigenesis [45].

### Anti-hepatocarcinoma activity

Various types of AC extracts and purified components (*e.g.*, antcin A, B, and C, antroquinonol, methyl antcinate A (MAA), methyl antcinate B (MAB) ethylacetate, and methanolic, ethanolic and fermented extracts) exhibit cytotoxicity through apoptosis in various cancers, especially hepatic carcinoma. Apoptosis is processed sequentially through the activation of death receptors (*e.g.*, Fas receptor) and downstream caspases (*e.g.*, caspase-3, -8, and -9), and mitochondrial function disruption. MAA (30.2–78 μM) and antcins A and C (30.2–286.4 μM) stimulate a series of apoptotic pathway cascades, including caspase-2, -3, and -9 activation, Bax, Bak, and PUMA protein expression, Bcl-(XL) and Bcl-2 suppression, and cytochrome c release, resulting in apoptosis [46-48]. MAB and antcin B (100 μM)-induced apoptosis is characterized by increased DNA fragmentation, cleavage of PARP, sub-G1 population appearance, and chromatin condensation [49]. Moreover, AC components induced NADPH oxidase-provoked oxidative stress [49,50]. Another purified AC component, antroquinonol, inhibited HepG2 (IC_50_: 0.13 μM) and Hep3B (IC_50_: 4.3 μM) cell growth [50,51]. Recently, Chiang et al. [52] demonstrated that antroquinonol induced HCC apoptosis through AMPK activation and mTOR translational pathway inhibition, and induced cell cycle arrest through downregulation and suppressed nuclear translocation of cyclin D1, cyclin E, Cdk4, and Cdk2. An AC ethyl acetate extract (IC_50_: 42.5 μg/mL in HepG2 cells; 78.3 μg/mL in Hep3B cells), methanolic extract (IC_50_: 49.5 μg/mL in HepG2 cells; 62.7 μg/mL in Hep3B cells), and ethanolic extract (IC_50_: 54.2 μg/mL in HepG2 cells; 82.9 μg/mL in Hep3B cells) could also inhibit growth in a similar manner [4,46,48,53,54]. Apart from the direct cytotoxicity, cell cycle arrest can be used for controlling tumor growth. During the G1 phase of the cell cycle, the cells increase in size and synthesize essential proteins for the resting steps of the cell division process. It is important to control whether a cell goes into the S phase (DNA synthesis phase) or escapes from the cell cycle. An AC mycelium extract, 4-acetylantroquinonol B (EC_50_: 0.1 μg/ mL), induced cell cycle arrest in HepG2 cells, indicating that cells were unable to divide. Western blotting analyses further showed that the protein expressions of different cell cycle-regulating proteins, CDK2 and CDK4, were decreased, while p21, p27, and p53 were increased in HepG2 cells after the AC treatment [55,56]. Similar to the case for the conventional anti-cancer drug cisplatin, tumor cell proliferation can be inhibited through induction of DNA damage, cell cycle arrest, and apoptosis. This suppresses the size of tumor tissues, and reduces the chance for subsequent metastasis [57,58].

Anti-angiogenic therapy has been explored as a therapeutic approach to HCC [59,60]. A series of angiogenic inhibitors, *e.g.*, bevacizumab (for blocking the angiogenic factor VEGF), endostatin, IFN-α, cyclooxygenase-2 (for inhibiting EC migration, tube formation, proliferation, and angiogenic factor production), interleukin-12 (for producing anti-angiogenic factors), and erlotinib and gefitinib (for inhibiting epidermal growth factor receptor tyrosine kinase activation) are currently in different phases of clinical trials [61].

An AC ethyl acetate extract and AC polysaccharides inhibited angiogenesis *in vitro* and *in vivo*. The ethyl acetate extract was shown to inhibit cancer invasion of human liver cancer PLC/PRF/5 cells through suppression of angiogenic factor (e.g. VEGF) and extracellular digestive enzyme (e.g. MMP-2 and -9) production, and elevation of enzyme inhibitor (tissue inhibitor of metalloproteinases (TIMP)-1 and TIMP-2) production in the concentration ranges of 10 to 40 μg/mL [47]. These results were confirmed in an animal model by Matrigel plug angiogenesis assays, in which the ethyl acetate extract (300 mg/kg) significantly suppressed tumor growth in nude mice [46]. The VGEF-induced tube formation and migration of ECs, and neovessel formation on chorioallantoic membranes were effectively inhibited by the AC polysaccharide extract [62,63]. Cheng et al. [64] demonstrated that the different fractionated AC polysaccharide extracts were able to inhibit VEGF protein expression, binding of VEGF to VEGFR-2, and VEGFR-2 phosphorylation, suggesting that inhibition of VEGF interactions with VEGF receptors is an anti-angiogenic mechanism of AC.

Anti-angiogenic therapy is particularly promising, because it is expected to minimize the devastating side effects, such as hair loss, bleeding, and immunosuppression, that are commonly associated with conventional chemotherapy. We previously found that many herbal medicines and natural compounds, such as ginsenosides, indirubin derivatives, and sinomenine, possess anti-angiogenic activities [65-70]. Therefore, AC may also contain anti-angiogenic components.

## Effects on alcohol metabolism

### Metabolism of alcohol and its related diseases

Chronic excessive consumption of alcohol often causes serious health problems, especially to the liver, such as cirrhosis and HCC [18,71]. According to the WHO, alcohol is the third largest risk factor for disease worldwide, and has been classified as a type I carcinogen by the IARC [72]. It has been estimated that alcohol leads to 2.5 million deaths each year, and that 320,000 of these deaths are in the age group of 15–29 years, representing 9% of all deaths in that age group [73]. Alcohol, a water-soluble small molecule, is easily absorbed into the bloodstream from the gastrointestinal tract. It cannot be stored, and is either metabolized or excreted [74,75]. More than 90% of absorbed alcohol is oxidized by hepatocytes, while the remainder is excreted through pulmonary respiration and urinary excretion [75,76]. There are three major pathways for metabolizing alcohol. The primary pathway of alcohol metabolism is the alcohol dehydrogenase (ADH)-mediated ethanol oxidation pathway [76,77]. ADH comprises a group of cytosolic enzymes that convert alcohol to acetaldehyde utilizing NAD^+^ as a coenzyme, and the elements of this pathway are mainly located in the liver. At high alcohol concentrations (above 100 mg/dL) or during chronic alcohol consumption, the ADH pathway may become saturated through NAD^+^ depletion. When this happens, the secondary pathway, the microsomal ethanol oxidizing system, is used to break down alcohol [76,77]. In this system, cytochrome P450 enzymes such as CYP2E1, CYP1A2, and CYP2A4 metabolize alcohol to acetaldehyde via NADPH as a coenzyme instead of NAD^+^[76,78]. The third pathway is the hydrogen peroxide (H_2_O_2_)-generating system in peroxisomes [76], which oxidizes alcohol to acetaldehyde and water molecules [75,77]. The acetaldehyde product is further oxidized by aldehyde dehydrogenase (ALDH) to acetate, and subsequently processed in the citric acid cycle. The final products are carbon dioxide, water, and ATP. Through the ADH and ALDH-mediated reactions, NAD^+^ is used up in the form of a coenzyme, and NADH is generated. This increased ratio of NADH:NAD^+^ redox potential in the liver inhibits mitochondrial β-oxidation of fatty acids and reduces lipid expenditure, resulting in lipogenesis and lipid accumulation in hepatocytes [79,80]. The severity of this alcoholic liver disease (ALD) depends on the amount, frequency, and duration of alcohol consumption, and can further develop into more complicated liver damage states, including fibrosis, steatohepatitis, cirrhosis, and cancer [81-83].

### Anti-alcoholic activity

Different types of AC preparations, including ethanolic extracts, aqueous extracts, fermented filtrates, and crude powder, can protect the liver against alcohol damage. Ethanol-induced ALD rats were orally treated with AC ethanolic extracts at a range of 0.25–1.25 g/kg. Hepatic damage was assessed using serum markers of liver damage, including aspartate aminotransferase (AST), alanine aminotransferase (ALT), and alkaline phosphatase (ALP) [84]. When liver cells are damaged, these enzymes are released into the bloodstream where they can be detected with biochemical or immunological assay kits. The AC extracts could prevent ethanol- and carbon tetrachloride (CCl_4_)-induced elevations of the AST, ALT, and ALP levels in the rat blood serum [85,86], indicating that AC conferred protection against chemical-induced liver damage. Moreover, the AC ethanolic extract could normalize the alcohol-induced increase in malondialdehyde, and decreases in glutathione peroxidase, reductase, and glutathione levels in the liver [86,87]. In the CCl_4_-induced ALD model, AC extracts could restore the reduction in the hepatic glutathione content and catalase (CAT) activity, and prevent free radical formation and lipid peroxidation [88]. These *in vivo* data were validated in a HepG2 cellular model by Kumar et al. [89], who showed that the antroquinonol from the AC ethanolic extract could protect ethanol-induced HepG2 cells against heme oxygenase 1, NF-E2 released factor (Nrf-2), and mitogen-activated protein kinase activation (MAPK).

An AC fermented filtrate protected HepG2 cells against damage caused by CCl_4_[90] and H_2_O_2_[91]. Song et al. [91] demonstrated that the AC fermented filtrate (0.05–0.5 mg/mL) suppressed lipid peroxidation in H_2_O_2_-induced HepG2 cells. The AC fermented filtrate restored the CCl_4_-induced increases in ALT, AST, lipid peroxidation, liver lesions, neutrophil infiltration, hydropic swelling, and necrosis, and the reductions in glutathione peroxidase, reductase, and transferase in animal tests [91]. The liver damage caused by chronic alcohol consumption was found to be reduced by an AC crude powder. Chen et al. [85] demonstrated that AC crude powder-treated rats had relatively smaller livers, less lipid accumulation, and lower AST, ALP, and serum alcohol levels. In addition, their serum and hepatic MMP-9 activities, and TNF-α, krueppel-like factor-6 (KLF-6), and transformation growth factor-β (TGF-β) gene expressions were downregulated. Moreover, the AC powder could enhance the metabolism of alcohol by increasing the CAT and ALDH activities [85]. Chen et al. [92] further demonstrated that the AC crude powder induced downregulation of 3-hydroxy-3-methylglutaryl-CoA reductase, sterol regulatory element-binding protein-1c, acetyl-CoA carboxylase, fatty acid synthase, and malic gene expression, and upregulation of low-density lipoprotein receptor and peroxisome proliferator-activated alpha gene expression [92]. A dried powder from fermented AC mycelium (0.34–0.57 g/kg) could protect the rat liver against damage from alcohol, and significantly suppress the alcohol-induced increases in glutamate-pyruvate aminotransferase, glutamate-oxaloacetate aminotransferase, superoxide dismutase, and CAT activities. Histological data validated the AC protective effects by showing a reduction in alcohol-induced lipid vacuole accumulation, and hydropic degradation of hepatocytes [93].

## Conclusions

Hepatoprotective activities, including anti-hepatitis, anti-hepatocarcinoma, and anti-alcoholism, of AC have been demonstrated *in vitro* and *in vivo*. However, many of the underlying mechanistic actions, such as the pharmacokinetics and pharmacodynamics, remain unclear. Furthermore, human and clinical trials are required for a full evaluation of this valuable medicinal mushroom for potential clinical applications.

## Abbreviations

AC: Antrodia cinnamomea; FDA: Food and Drug Administration; HAV: Hepatitis A virus; HBV: Hepatitis B virus; HCV: Hepatitis C virus; HDV: Hepatitis D virus; HCC: Hepatocellular carcinoma; HBsAg: Hepatitis B surface antigen; HBeAg: Hepatitis B e antigen; IFN-α: Interferon-α; WHO: World Health Organization; IARC: International Agency for Research on Cancer; TNF-α: Tumor necrosis factor; IL-1β: Interleukin-1β; IL-6: Interleukin-6; VEGF: Vascular endothelial cell growth factor; FGF-2: Fibroblast growth factor-2; EC: Endothelial cell; VEGFR-1: VEGF receptor 1; VEGFR-2: VEGF receptor 2; MMP: Matrix metalloproteinase; MAA: Methyl antcinate A; MAB: Methyl antcinate B; TIMP-1: Tissue inhibitor of metalloproteinases-1; TIMP-2: Tissue inhibitor of metalloproteinases-2; ADH: Alcohol dehydrogenase; H2O2: Hydrogen peroxide; ALDH: Aldehyde dehydrogenase; ALD: Alcoholic liver disease; AST: Aspartate aminotransferase; ALT: Alanine aminotransferase; ALP: Alkaline phosphatase; CCl4: Carbon tetrachloride; CAT: Catalase; Nrf-2: NF-E2 released factor; MAPK: Mitogen-activated protein kinase activation; ALDH: Aldehyde dehydrogenase.

## Competing interests

The authors declare that they have no competing interest.

## Authors' contributions

PYKY was responsible for drafting and proofreading of this article. The information searching was done by YYW, KYKW, and YKT. Final proofreading was done by KSYL. All authors read and approved the final manuscript.

## References

[B1] Chen CJ Su CH Lan MH Study on solid cultivation and bioactivity of Antrodia camphortaa Fungal Sci 2001 16 65 72

[B2] Zang M Su CH *Ganoderma comphoratum*, a new taxon in genus *Ganderma* from Taiwan, China Acta Bot Yunnanica 1990 12 395 396

[B3] Phuong do T Ma CM Hattori M Jin JS Inhibitory effects of antrodins A-E from *Antrodia cinnamomea* and their metabolites on hepatitis C virus protease Phytother Res 2009 23 582 584 10.1002/ptr.2657 19003946

[B4] Chen YS Pan JH Chiang BH Lu FJ Sheen LY Ethanolic extracts of *Antrodia cinnamomea* mycelia fermented at varied times and scales have differential effects on hepatoma cells and normal primary hepatocytes J Food Sci 2008 73 H179 H185 10.1111/j.1750-3841.2008.00884.x 18803715

[B5] Ryder SD Beckingham IJ ABC of diseases of liver, pancreas, and biliary system: acute hepatitis BMJ 2001 322 151 153 10.1136/bmj.322.7279.151 11159575 PMC1119417

[B6] Matheny SC Kingery JE Hepatitis A Am Fam Physician 2012 86 1027 1034 23198670

[B7] Conjeevaram HS Lok AS Management of chronic hepatitis B J Hepatol 2003 38 S90 S103 12591188 10.1016/s0168-8278(02)00431-2

[B8] Perrillo RP Management of the patient with hepatitis B virus-related cirrhosis J Hepatol 2003 39 S177 S180 14708700 10.1016/s0168-8278(03)00320-9

[B9] Chisari FV Rous-Whipple award lecture. Viruses, immunity, and cancer: lessons from hepatitis B Am J Pathol 2000 156 1117 1132 10.1016/S0002-9440(10)64980-2 10751335 PMC1876872

[B10] Mulkay JP Hepatitis B: screening and treatment Rev Med Brux 2012 33 215 222 23091924

[B11] Peppa D Maini MK Pathogenesis of hepatitis B virus infection and potential for new therapies Br J Hosp Med (Lond) 2012 73 581 584 23124289 10.12968/hmed.2012.73.10.581

[B12] Cohen J Report of new hepatitis virus has researchers intrigued and upset Science 1999 285 644 645 10.1126/science.285.5428.644 10454907

[B13] Cohen J The scientific challenge of hepatitis C Science 1999 285 26 30 10.1126/science.285.5424.26 10428695

[B14] Terilli RR Cox AL Immunity and hepatitis C: a review Curr HIV/AIDS Rep 2013 10 51 58 10.1007/s11904-012-0146-4 23180007 PMC3567217

[B15] Bouchard MJ Navas-Martin S Hepatitis B and C virus hepatocarcinogenesis: lessons learned and future challenges Cancer Lett 2011 305 123 143 10.1016/j.canlet.2010.11.014 21168955 PMC3071446

[B16] Lee IH Huang RL Chen CT Chen HC Hsu WC Lu MK *Antrodia camphorata* polysaccharides exhibit anti-hepatitis B virus effects FEMS Microbiol Lett 2002 209 63 67 10.1111/j.1574-6968.2002.tb11110.x 12007655

[B17] Huang RL Huang QL Chen CF Chang TT Chou CJ Anti-viral effects of active compounds from *Antrodia camphorata* on wild-type and lamivudine-resistant mutant HBV Chin Pharmaceut J 2003 55 371 379

[B18] Popper H Thung SN Gerber MA Pathology of alcoholic liver diseases Semin Liver Dis 1981 1 203 216 10.1055/s-2008-1041748 6287646

[B19] Schaff Z Székely E Járay B Kiss A Pathology of liver tumors Orv Hetil 2004 145 368 374 15049053

[B20] Parkin DM Bray F Ferlay J Pisani P Estimating the world cancer burden: globocan 2000 Int J Canc 2001 94 153 156 10.1002/ijc.1440 11668491

[B21] Parkin DM Bray FI Devesa SS Cancer burden in the year 2000. The global picture Eur J Canc 2001 37 S4 S66 10.1016/s0959-8049(01)00267-2 11602373

[B22] Chen JG Zhu J Parkin DM Zhang YH Lu JH Zhu YR Chen TY Trends in the incidence of cancer in Qidong, China. 1978-2002 Int J Canc 2006 119 1447 1454 10.1002/ijc.21952 16596645

[B23] Deuffic S Poynard T Buffat L Valleron AJ Trends in primary liver cancer Lancet 1998 351 214 215 9449893 10.1016/S0140-6736(05)78179-4

[B24] El-Serag HB Mason AC Rising incidence of hepatocellular carcinoma in the United States N Engl J Med 1999 340 745 750 10.1056/NEJM199903113401001 10072408

[B25] El-Serag HB Davila JA Petersen NJ McGlynn KA The continuing increase in the incidence of hepatocellular carcinoma in the United States: an update Ann Intern Med 2003 139 817 823 10.7326/0003-4819-139-10-200311180-00009 14623619

[B26] Taylor-Robinson SD Foster GR Arora S Hargreaves S Thomas HC Increase in primary liver cancer in the UK, 1979-94 Lancet 1997 350 1142 1143 9343506 10.1016/S0140-6736(05)63789-0

[B27] Wong CM Ng IO Molecular pathogenesis of hepatocellular carcinoma Liver Int 2008 28 160 174 18069974 10.1111/j.1478-3231.2007.01637.x

[B28] Idilman R De Maria N Colantoni A Van Thiel DH Pathogenesis of hepatitis B and C-induced hepatocellular carcinoma J Viral Hepat 1998 5 285 299 10.1046/j.1365-2893.1998.00116.x 9795912

[B29] WHO Surveillance and response: WHO. Hepatitis B Department of communable diseases 2002

[B30] Moradpour D Blum HE Pathogenesis of hepatocellular carcinoma Eur J Gastroenterol Hepatol 2005 17 477 483 10.1097/00042737-200505000-00002 15827436

[B31] Gomaa AI Khan SA Toledano MB Waked SD Taylor-Robinson, hepatocellular carcinoma: epidemiology, risk factors and pathogenesis World J Gastroenterol 2008 14 4300 4308 10.3748/wjg.14.4300 18666317 PMC2731180

[B32] Farazi PA DePinho RA Hepatocellular carcinoma pathogenesis: from genes to environment Nat Rev Canc 2006 6 674 687 10.1038/nrc1934 16929323

[B33] Bressac B Kew M Wands J Ozturk M Selective G to T mutations of p53 gene in hepatocellular carcinoma from southern Africa Nature 1991 350 429 431 10.1038/350429a0 1672732

[B34] Riley J Mandel HG Sinha S Judah DJ Neal GE *In vitro* activation of the human Harvey-ras proto-oncogene by aflatoxin B1 Carcinogenesis 1997 18 905 910 10.1093/carcin/18.5.905 9163674

[B35] Aguilar F Harris CC Sun T Hollstein M Cerutti P Geographic variation of p53 mutational profile in nonmalignant human liver Science 1994 264 1317 1319 10.1126/science.8191284 8191284

[B36] Ozturk M p53 mutation in hepatocellular carcinoma after aflatoxin exposure Lancet 1991 338 1356 1359 10.1016/0140-6736(91)92236-U 1682737

[B37] Hoek JB Pastorino JG Ethanol, oxidative stress, and cytokine-induced liver cell injury Alcohol 2002 27 63 68 10.1016/S0741-8329(02)00215-X 12062639

[B38] McClain CJ Hill DB Song Z Deaciuc I Barve S Monocyte activation in alcoholic liver disease Alcohol 2002 27 53 61 10.1016/S0741-8329(02)00212-4 12062638

[B39] Campbell JS Hughes SD Gilbertson DG Palmer TE Holdren MS Haran AC Odell MM Bauer RL Ren HP Haugen HS Yeh MM Fausto N Platelet-derived growth factor C induces liver fibrosis, steatosis, and hepatocellular carcinoma Proc Natl Acad Sci USA 2005 102 3389 3394 10.1073/pnas.0409722102 15728360 PMC552940

[B40] Parikh S Hyman D Hepatocellular cancer: a guide for the internist Am J Med 2007 120 194 202 10.1016/j.amjmed.2006.11.020 17349437

[B41] Ribatti D Vacca A Dammacco F The role of the vascular phase in solid tumor growth: a historical review Neoplasia 1999 1 293 302 10.1038/sj.neo.7900038 10935483 PMC1508099

[B42] Semela D Dufour JF Angiogenesis and hepatocellular carcinoma J Hepatol 2004 41 864 880 10.1016/j.jhep.2004.09.006 15519663

[B43] Bergers G Benjamin LE Tumorigenesis and the angiogenic switch Nat Rev Canc 2003 3 401 410 10.1038/nrc1093 12778130

[B44] Carmeliet P Jain RK Angiogenesis in cancer and other diseases Nature 2000 407 249 257 10.1038/35025220 11001068

[B45] Sugimachi K Tanaka S Terashi T Taguchi K Rikimaru T Sugimachi K The mechanisms of angiogenesis in hepatocellular carcinoma: angiogenic switch during tumor progression Surgery 2002 131 S135 S141 10.1067/msy.2002.119365 11821800

[B46] Hsu YL Kuo YC Kuo PL Ng LT Kuo YH Lin CC Apoptotic effects of extract from *Antrodia camphorata* fruiting bodies in human hepatocellular carcinoma cell lines Canc Lett 2005 221 77 89 10.1016/j.canlet.2004.08.012 15797630

[B47] Hsu YL Kuo PL Cho CY Ni WC Tzeng TF Ng LT Kuo YH Lin CC *Antrodia cinnamomea* fruiting bodies extract suppresses the invasive potential of human liver cancer cell line PLC/PRF/5 through inhibition of nuclear factor kappaB pathway Food Chem Toxicol 2007 45 1249 1257 10.1016/j.fct.2007.01.005 17316946

[B48] Kuo PL Hsu YL Cho CY Ng LT Kuo YH Lin CC Apoptotic effects of Antrodia cinnamomea fruiting bodies extract are mediated through calcium and calpain-dependent pathways in Hep 3B cells Food Chem Toxicol 2006 44 1316 1326 10.1016/j.fct.2006.02.009 16600460

[B49] Hsieh YC Rao YK Whang-Peng J Huang CY Shyue SK Hsu SL Tzeng YM Antcin B and its ester derivative from *Antrodia camphorata* induce apoptosis in hepatocellular carcinoma cells involves enhancing oxidative stress coincident with activation of intrinsic and extrinsic apoptotic pathway J Agric Food Chem 2011 59 10943 110954 10.1021/jf202771d 21916504

[B50] Hsieh YC Rao YK Wu CC Huang CY Geethangili M Hsu SL Tzeng YM Methyl antcinate A from Antrodia camphorata induces apoptosis in human liver cancer cells through oxidant-mediated cofilin- and Bax-triggered mitochondrial pathway Chem Res Toxicol 2010 23 1256 1267 10.1021/tx100116a 20557081

[B51] Lee TH Lee CK Tsou WL Liu SY Kuo MT Wen WC A new cytotoxic agent from solid-state fermented mycelium of *Antrodia camphorate* Planta Med 2007 73 1412 1415 10.1055/s-2007-990232 17932820

[B52] Chiang PC Lin SC Pan SL Kuo CH Tsai IL Kuo MT Wen WC Chen P Guh JH Antroquinonol displays anticancer potential against human hepatocellular carcinoma cells: a crucial role of AMPK and mTOR pathways Biochem Pharmacol 2010 79 162 171 10.1016/j.bcp.2009.08.022 19723512

[B53] Song TY Hsu SL Yen GC Induction of apoptosis in human hepatoma cells by mycelia of *Antrodia camphorata* in submerged culture J Ethnopharmacol 2005 100 158 167 10.1016/j.jep.2005.02.043 15949907

[B54] Song TY Hsu SL Yeh CT Yen GC Mycelia from *Antrodia camphorata* in Submerged culture induce apoptosis of human hepatoma HepG2 cells possibly through regulation of Fas pathway J Agric Food Chem 2005 53 5559 5564 10.1021/jf050329+ 15998114

[B55] Lin YW Chiang BH 4-acetylantroquinonol B isolated from *Antrodia cinnamomea* arrests proliferation of human hepatocellular carcinoma HepG2 cell by affecting p53, p21 and p27 levels J Agric Food Chem 2011 59 8625 8631 10.1021/jf2011326 21739974

[B56] Lin YW Pan JH Liu RH Kuo YH Sheen LY Chiang BH The 4-acetylantroquinonol B isolated from mycelium of *Antrodia cinnamomea* inhibits proliferation of hepatoma cells J Sci Food Agric 2010 90 1739 1744 10.1002/jsfa.4010 20564437

[B57] Cepeda V Fuertes MA Castilla J Alonso C Quevedo C Pérez JM Biochemical mechanisms of cisplatin cytotoxicity Anticancer Agents Med Chem 2007 7 3 18 10.2174/187152007779314044 17266502

[B58] Jordan P Carmo-Fonseca M Molecular mechanisms involved in cisplatin cytotoxicity Cell Mol Life Sci 2000 57 1229 1235 10.1007/PL00000762 11028915 PMC11147023

[B59] Ribatti D Vacca A Nico B Sansonno D Dammacco F Angiogenesis and anti-angiogenesis in hepatocellular carcinoma Canc Treat Rev 2006 32 437 444 10.1016/j.ctrv.2006.06.002 16870349

[B60] Pang R Poon RT Angiogenesis and antiangiogenic therapy in hepatocellular carcinoma Canc Lett 2006 242 151 167 10.1016/j.canlet.2006.01.008 16564617

[B61] Finn RS Zhu AX Targeting angiogenesis in hepatocellular carcinoma: focus on VEGF and bevacizumab Expert Rev Anticancer Ther 2009 9 503 509 10.1586/era.09.6 19374603

[B62] Chen SC Lu MK Cheng JJ Wang DL Antiangiogenic activities of polysaccharides isolated from medicinal fungi FEMS Microbiol Lett 2005 249 247 254 10.1016/j.femsle.2005.06.033 16046081

[B63] Yang CM Zhou YJ Wang RJ Hu ML Anti-angiogenic effects and mechanisms of polysaccharides from *Antrodia cinnamomea* with different molecular weights J Ethnopharmacol 2009 123 407 412 10.1016/j.jep.2009.03.034 19501273

[B64] Cheng JJ Huang NK Chang TT Wang DL Lu MK Study for anti-angiogenic activities of polysaccharides isolated from *Antrodia cinnamomea* in endothelial cells Life Sci 2005 76 3029 3042 10.1016/j.lfs.2004.11.023 15850596

[B65] Chan YK Kwok HH Chan LS Leung KS Shi J Mak NK Wong RN Yue PY An indirubin derivative, E804, exhibits potent angiosuppressive activity Biochem Pharmacol 2012 83 598 607 10.1016/j.bcp.2011.12.003 22178720

[B66] Yue PY Mak NK Cheng YK Leung KW Ng TB Fan DT Yeung HW Wong RN Pharmacogenomics and the Yin/Yang actions of ginseng: anti-tumor, angiomodulating and steroid-like activities of ginsenosides Chin Med 2007 2 42 62 10.1186/1749-8546-2-6 PMC1876803 17502003

[B67] Leung KW Yung KK Mak NK Yue PY Luo HB Cheng YK Fan TP Yeung HW Ng TB Wong RN Angiomodulatory and neurological effects of ginsenosides Curr Med Chem 2007 14 1371 1380 10.2174/092986707780597916 17504218

[B68] Yue PY Wong DY Wu PK Leung PY Mak NK Yeung HW Liu L Cai Z Jiang ZH Fan TP Wong RN The angiosuppressive effects of 20(R)-ginsenoside Rg3 Biochem Pharmacol 2006 72 437 445 10.1016/j.bcp.2006.04.034 16793023

[B69] Fan TP Yeh JC Leung KW Yue PY Wong RN Angiogenesis: from plants to blood vessels Trends Pharmacol Sci 2006 27 297 309 10.1016/j.tips.2006.04.006 16697473

[B70] Kok TW Yue PY Mak NK Fan TP Liu L Wong RN The anti-angiogenic effect of sinomenine Angiogenesis 2005 8 3 12 10.1007/s10456-005-2892-z 16132613

[B71] Charels K Klöppel G Pathology and pathogenesis of toxic (alcoholic) liver disease Hepatogastroenterology 1991 38 189 194 1855777

[B72] IARC Working Group on the Evaluation of Carcinogenic Risks to Humans Personal habits and indoor combustions. Volume 100 E. A review of human carcinogens IARC Monogr Eval Carcinog Risks Hum 2012 100 1 538 PMC4781577 23193840

[B73] Yip WW Burt AD Alcoholic liver disease Semin Diagn Pathol 2006 23 149 160 10.1053/j.semdp.2006.11.002 17355088

[B74] Doria JJ Alcohol metabolism Alcohol Health Res World 1997 21 323

[B75] Bode C Bode JC Alcohol absorption, metabolism, and production in the gastrointestinal tract Alcohol Health Res World 1997 21 82 83 PMC6826790 15706765

[B76] Zakhari S Overview: How is alcohol metabolized by the body? Alcohol Res Health 2006 29 245 254 17718403 PMC6527027

[B77] Lieber CS Hepatic, metabolic and toxic effects of ethanol: 1991 update Alcohol Clin Exp Res 1991 15 573 592 10.1111/j.1530-0277.1991.tb00563.x 1928631

[B78] Lieber C The discovery of the microsomal ethanol oxidizing system and its physiologic and pathologic role Drug Metab Rev 2004 36 511 529 10.1081/DMR-200033441 15554233

[B79] Donohue TM Alcohol-induced steatosis in liver cells World J Gastroenterol 2007 13 4974 4978 17854140 10.3748/wjg.v13.i37.4974 PMC4434621

[B80] Sozio M Crabb DW Alcohol and lipid metabolism Am J Physiol Endocrinol Metab 2008 295 E10 E16 10.1152/ajpendo.00011.2008 18349117 PMC2493591

[B81] Bruha R Dvorak K Petrtyl J Alcoholic liver disease World J Hepatol 2012 4 81 90 10.4254/wjh.v4.i3.81 22489260 PMC3321494

[B82] Syn WK Teaberry V Choi SS Diehl AM Similarity and differences in the pathogenesis of alcoholic and non-alcoholic steatohepatiotis Semin Liver Dis 2009 29 200 210 10.1055/s-0029-1214375 19387919 PMC3644873

[B83] Fernández-Checa JC Kaplowitz N Colell A García-Ruiz C Oxidative stress and alcoholic liver disease Alcohol Health Res World 1997 21 321 324 15706743 PMC6827680

[B84] Lieber CS Alcohol, liver, and nutrition J Am Coll Nutr 1991 10 602 632 10.1080/07315724.1991.10718182 1770192

[B85] Wu MT Tzang BS Chang YY Chiu CH Kang WY Huang CH Chen YC Effects of *Antrodia camphorata* on alcohol clearance and antifibrosis in livers of rats continuously fed alcohol J Agric Food Chem 2011 59 4248 4254 10.1021/jf104561h 21401100

[B86] Lu ZM Tao WY Zou XL Fu HZ Ao ZH Protective effects of mycelia of *Antrodia camphorata* and *Armillariella tabescens* in submerged culture against ethanol-induced hepatic toxicity in rats J Ethnopharmacol 2007 110 160 164 10.1016/j.jep.2006.09.029 17092673

[B87] Lu ZM Tao WY Xu HY Ao ZH Zhang XM Xu ZH Further studies on the hepatoprotective effect of *Antrodia camphorata* in submerged culture on ethanol-induced acute liver injury in rats Nat Prod Res 2011 25 684 695 10.1080/14786410802525487 20623423

[B88] Hsiao G Shen MY Lin KH Lan MH Wu LY Chou DS Lin CH Su CH Sheu JR Antioxidative and hepatoprotective effects of *Antrodia camphorata* extract J Agric Food Chem 2003 51 3302 3308 10.1021/jf021159t 12744658

[B89] Kumar KJ Chu FH Hsieh HW Liao JW Li WH Lin JC Shaw JF Wang SY Antroquinonol from ethanolic extract of mycelium of *Antrodia cinnamomea* protects hepatic cells from ethanol-induced oxidative stress through Nrf-2 activation J Ethnopharmacol 2011 136 168 177 10.1016/j.jep.2011.04.030 21540101

[B90] Lin WC Kuo SC Lin WL Fang HL Wang BC Filtrate of fermented mycelia from *Antrodia camphorata* reduces liver fibrosis induced by carbon tetrachloride in rats World J Gastroenterol 2006 12 2369 2374 16688827 10.3748/wjg.v12.i15.2369 PMC4088072

[B91] Song TY Yen GC Protective effects of fermented filtrate from *Antrodia camphorata* in submerged culture against CCl4-induced hepatic toxicity in rats J Agric Food Chem 2003 51 1571 1577 10.1021/jf0209701 12617586

[B92] Huang CH Chang YY Liu CW Kang WY Lin YL Chang HC Chen YC Fruiting body of Niuchangchih (*Antrodia camphorata*) protects livers against chronic alcohol consumption damage J Agric Food Chem 2010 58 3859 3866 10.1021/jf100530c 20192205

[B93] Dai YY Chuang CH Tsai CC Sio HM Huang SC Chen JC Hu ML The proliferation of *Anthrodia camphorate* against acute hepatotoxicity of alcohol in rat J Food Drug Ann 2003 11 177 185

